# Benzodiazepine Interference with Fertility and Embryo Development: A Preliminary Survey in the Sea Urchin *Paracentrotus lividus*

**DOI:** 10.3390/ijms25041969

**Published:** 2024-02-06

**Authors:** Chiara Fogliano, Rosa Carotenuto, Paola Cirino, Raffaele Panzuto, Martina Ciaravolo, Palma Simoniello, Ilaria Sgariglia, Chiara Maria Motta, Bice Avallone

**Affiliations:** 1 Department of Biology, University of Naples Federico II, 80126 Naples, Italy; chiara.fogliano@unina.it (C.F.); rosa.carotenuto@unina.it (R.C.); martinaciaravolo@gmail.com (M.C.); i.sgariglia@studenti.unina.it (I.S.); bice.avallone@unina.it (B.A.); 2 Department of Conservation of Marine Animals and Public Engagement, Anton Dohrn Zoological Station, 80122 Naples, Italy; paola.cirino@szn.it (P.C.); raffaele.panzuto@szn.it (R.P.); 3 Department of Science and Technology, University of Naples Parthenope, 80133 Naples, Italy; palma.simoniello@uniparthenope.it

**Keywords:** fertility rate, gamete quality, pluteus development, lectin staining, gut development

## Abstract

Psychotropic drugs and benzodiazepines are nowadays among the primary substances of abuse. This results in a large and constant release into aquatic environments where they have potentially harmful effects on non-target organisms and, eventually, human health. In the last decades, evidence has been collected on the possible interference of benzodiazepines with reproductive processes, but data are few and incomplete. In this study, the possible negative influence of delorazepam on fertilization and embryo development has been tested in *Paracentrotus lividus*, a key model organism in studies of reproduction and embryonic development. Sperm, eggs, or fertilized eggs have been exposed to delorazepam at three concentrations: 1 μg/L (environmentally realistic), 5 μg/L, and 10 μg/L. Results indicate that delorazepam reduces the fertilizing capacity of male and female gametes and interferes with fertilization and embryo development. Exposure causes anatomical anomalies in plutei, accelerates/delays development, and alters the presence and distribution of glycoconjugates such as N-Acetyl-glucosamine, α-linked fucose, and α-linked mannose in both morulae and plutei. These results should attract attention to the reproductive fitness of aquatic species exposed to benzodiazepines and pave the way for further investigation of the effects they may exert on human fertility. The presence of benzodiazepines in the aquatic environment raises concerns about the reproductive well-being of aquatic species. Additionally, it prompts worries regarding potential impacts on human fertility due to the excessive use of anxiolytics.

## 1. Introduction

In recent decades, surface and deep-water pollution has become a problem of great concern. The presence of contaminants of anthropogenic origin is an issue for aquatic species but also for humans who use contaminated waters directly for feeding, drinking, and breeding. For this reason, for over two decades, the presence and effects of Pharmaceutically Active Compounds (PhACs) in aquatic systems have been the subject of increasing scientific attention [1,2,3,4]. They are constantly released into the environment through routine pharmaceutical use by humans, farms, health facilities, and pharmaceutical industries and are not efficiently demolished by wastewater treatment plants (WWTPs) [2,4]. In recent years, and especially after the COVID-19 pandemic, the consumption of psychoactive pharmaceuticals (PaPs), anxiolytics, and antidepressants has increased worldwide, and so has the release into the environment [4,5].

Benzodiazepines (BZDs) are now among the most abused drugs and are frequently found as contaminants in the aquatic environment [6,7,8,9,10]. Many different types may be present simultaneously, at concentrations ranging from ng/L up to 0.89 μg/L in rivers (diazepam [11,12]) and up to 117 ng/L in seawater (lorazepam [8]). They differ in pharmacokinetic characteristics and absorption, sometimes forming very active metabolites, with long half-lives and prolonged activity [13]. Evidence indicates the cytotoxic effects of BZDs on non-target aquatic species: for example, in *Mytilus galloprovincialis*, delorazepam (DLZ) alters gill anatomy, causes goblet cell hyperplasia, and alters protein metabolism, also interfering with the presence and/or distribution of gill glycoconjugates [7]. In *Xenopus laevis* embryos, numerous tissues are affected after treatment with DLZ, such as the retina, skeletal muscle, or intestine, leading to both structural and functional damage [14].

Benzodiazepines also interfere with reproduction, affecting not only reproductive behavior, but also male and female gametes [15,16]. These effects have been described, for example, in bony fish [17], crustaceans (*Daphnia* [18]; *Mysidopsis* [19]), and gastropods (*Radix balthica* [20]). In the embryonic stages, DLZ is embryotoxic, teratogenic, and epigenotoxic, and dysmorphogenesis is accompanied by relevant changes in glycoconjugate distribution [6,14]. The side effects of BZD treatment on reproduction occur also in mammals; administration in utero has been associated with anatomical defects [21] and risks of preterm birth and low birth weight [22,23].

Based on these premises, we studied the effects of DLZ, a high half-life BZD [24], on sea urchin early development with particular attention to glycoconjugate distribution in morulae (6 hours post fertilization, hpf) and plutei (48 hpf). DLZ is one of the benzodiazepines with the highest elimination half-life (80–115 h). It is both a metabolite of diazepam and a parent molecule, marketed as a drug, and produces a major active metabolite known as lorazepam [6,24]. *Paracentrotus lividus* is an excellent experimental model for studies on development, since its spawning and fertilization occur in water, its development is fast, and it produces a transparent planktonic larva, the pluteus. The morphological anomalies induced by exposure to stressors represent an easy biomarker in ecotoxicological investigations [25,26,27,28]. For these reasons, the sea urchin is indicated by the European Agency for Alternative Models for studies on reproduction and toxicity testing [29], fully in line with the 3Rs objectives [30]. In addition, testing on benthonic echinoderms has environmental relevance since the sea urchin is a key species in coastal marine ecosystems [31] and, therefore, a good bioindicator organism [32,33]. 

The present study aimed to determine the effects of an environmentally realistic concentration of DLZ (1 µg/L [6,7]) and, in parallel, of two higher doses (5 and 10 µg/L), in consideration of the growing consumption trend, favored by the post-pandemic scenario [34,35]. Four different experiments were carried out to detect the interference of the drug on eggs and sperm quality, fertilization, and development. Fertilization rate, larval development, and anatomy were verified at 6 (morula stage) and 48 (pluteus stage) hours post fertilization. Considering the evidence that DLZ interferes with glycoconjugate distributions in two different species [7,14], a panel of five FITC-lectins was used to detect possible changes in the presence and localization of terminal N-Acetyl glucosamine (WGA), α-linked mannose (Con A) or fucose (UEA-I), and α- or β-linked N-Acetyl galactosamine (DBA and SBA) [36]. Particular attention was dedicated to spicules, affected by several toxicants [37], and to the gut, one of the target organs of DLZ in *Xenopus laevis* embryos [6] and, possibly, also in mammals [38].

## 2. Results

### 2.1. Effects of DLZ on Fertilization

**Figure 1:**
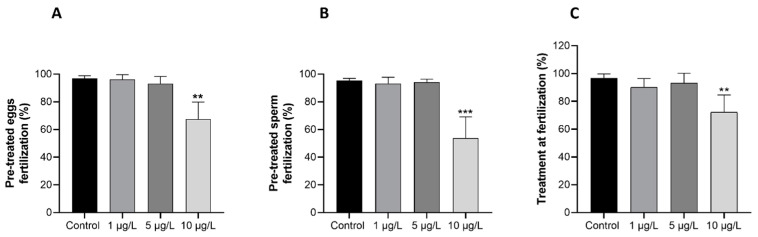
Effects of DLZ on fertilization percentage in the sea urchin *Paracentrotus lividus*. (**A**) Pre-treated eggs. (**B**) Pre-treated sperm. (**C**) Treatment at fertilization. Significant effects are registered exclusively at the higher dose. (**, *p* < 0.01; ***, *p* < 0.001).

Twenty minutes after fertilization, in control samples, 97.9 ± 1.2% of the eggs showed a fertilization membrane. In samples obtained from pre-treated eggs (Figure 1A), pre-treated sperm (Figure 1B), or gametes treated at fertilization (Figure 1C), no effects were registered on fertilization percentage at 1 or 5 µg/L DLZ (fertilization percentages ranging from a minimum of 90.1 to a maximum of 96.3%). In contrast, in samples treated with 10 µg/L DLZ, the average fertilization percentage was significantly reduced (*p* < 0.01), with values dropping to 67.5 ± 12.4%, 53.8 ± 15.4%, and 72.2 ± 12.3%, respectively.

### 2.2. Effects of DLZ on Morulae Development (6 hpf)

**Figure 2:**
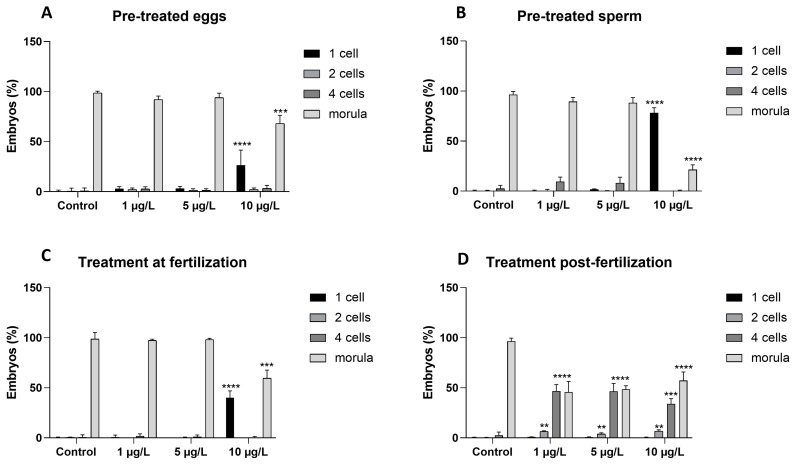
Effects of DLZ on cleavage progression: percentage of morulae six hours post fertilization (hpf). (**A**) Pre-treated eggs. (**B**) Pre-treated sperm. (**C**) Treatment at fertilization. (**D**) Treatment after fertilization. Significant effects are registered only at the higher dose, in (**A**–**C**). In sample (**D**), DLZ significantly delays cleavage at all tested concentrations. (**, *p* < 0.01; ***, *p* < 0.001; ****, *p* < 0.0001 with respect to control).

In control samples, six hours post fertilization (hpf), 97.6 ± 1.7 of the eggs were at the morula stage (Figure 2A–D). These were composed of 32–64 cells, with an incipient central cavity, the blastocoel. Pre-treatments of the eggs (Figure 2A) or the sperm (Figure 2B), or exposure to DLZ at fertilization (Figure 2C) caused no significant effects at the two lower DLZ concentrations. Percentages ranged from a minimum of 88.3 to a maximum of 98%. In contrast, exposure to 10 µg/L caused a very significant reduction in the percentage of morulae, with values ranging between a maximum of 68.1 and a minimum of 57.3% (Figure 2A–C). The effect size for egg pre-treatment was 2.2 at 1 µg/L, 1.6 at 5 µg/L, and >10 at 10 µg/L. For sperm pre-treatment, the minimum effect size was around 2.2 (at 1 and 5 µg/L, respectively) and >10 at 10 µg/L. For treatment at fertilization, no significant effect size was reported at 5 µg/L, while for 1 µg/L and 10 µg/L, the effect sizes were 0.9 and >10, respectively.

In samples treated after fertilization, the situation was different. At all concentrations, the percentage of morulae was drastically reduced from a minimum of 45.8% in the 1 µg/L sample to a maximum of 57.3% in 10 µg/L. The remaining embryos were delayed, still at the four-cell stage (from 33.9% in 10 µg/L to a maximum of 46.8 in 5 µg/L) or the two-cell stage (from 3.8 in 5 µg/L to 6.7% in 10 µg/L). A large effect size (>10) was reported at all concentrations.

### 2.3. Effects of DLZ on Plutei Development (48 hpf)

**Figure 3:**
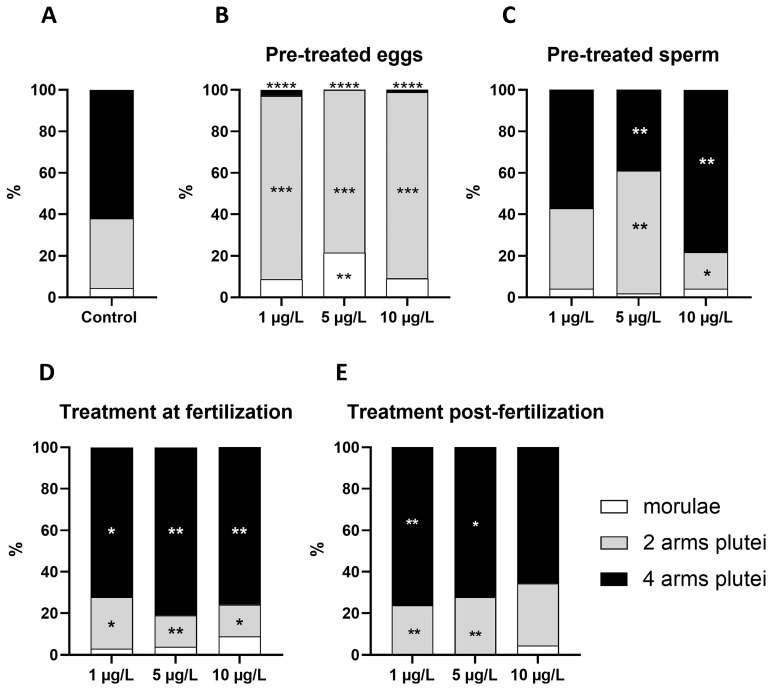
Effect of DLZ on the composition of the population of plutei. (**A**) 4-arm plutei represent about two-thirds of the individuals. The remaining one-third is composed of two-arm plutei and occasional morulae. (**B**–**E**) Significant variation in population composition. (*, *p* < 0.5; **, *p* < 0.01; ***, *p* < 0.001; ****, *p* < 0.0001 with respect to control).

In controls, at this time, the larvae developed a pair of post-oral arms and a pair of short pre-oral arms. On average, 34% of the plutei were at the two-arm stage (only post-oral arms developed) and 62% at the four-arm stage (Figure 3A); occasional unfertilized eggs (less than 4%) were observed. In the samples obtained from pre-treated eggs (Figure 3B), plutei were late, since four-arm plutei were very rare—no more than 2.8% of the population. The two-arm plutei represented the most consistent part of the population, ranging from a minimum of 78.6% in 5 µg/L samples to a maximum of 89.9% in 10 µg/L samples. A large effect size (>10) was reported. In samples obtained from pre-treated sperm (Figure 3C), the only treatment resembling control was DLZ 1 µg/L, in which 57.1% of plutei were at the four-arm stage and the effect size was 1.4. In samples exposed to 5 or 10 µg/L, the percentage of four-arm plutei was either significantly smaller (38.9%, 5 µg/L) or significantly higher (78.2, 10 µg/L), and the effect size >5. Both treatments at fertilization (Figure 3D) or after fertilization (Figure 3E) tended to cause a significant increase in the percentage of four-arm plutei (*p* < 0.01). Values varied from a minimum of 63.0% to a maximum of 81.1%. For these samples, the minimum effect size was 3. Morulae were also observed in all samples, the higher percentage being found in samples obtained from pre-treated eggs (from 8.9 to 21.4% of the entire population of embryos).

**Figure 4:**
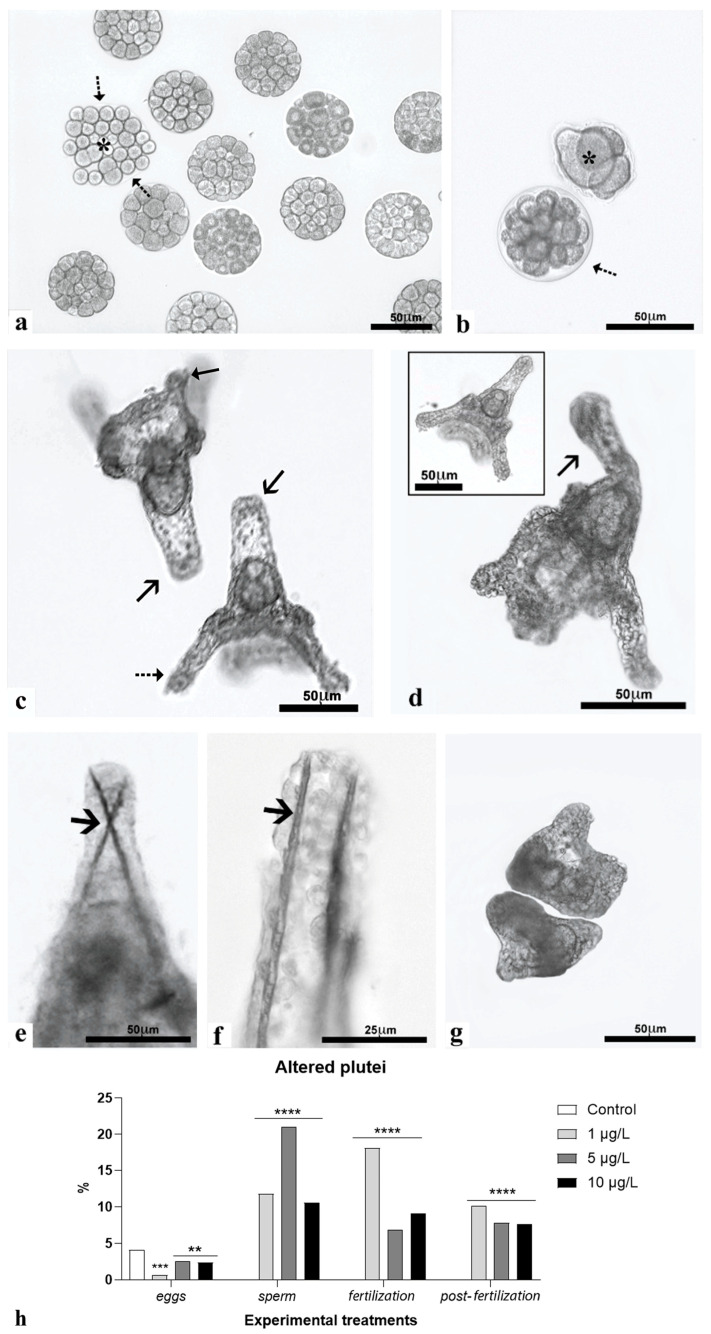
Effects of DLZ on larval morphology. (**a**) Morula with dissociated blastomeres (*) in a fertilization membrane (dotted arrows). (**b**) Abnormally developed morula (*). Fertilization membrane (dotted arrow). (**c**) Plutei with enlarged tip of the anterior arm (arrows). Pre- (small arrow) and post- (dotted arrow) oral arms. (**d**) Pluteus with bent arm (arrow). Inset: intact pluteus. (**e**,**f**) Detail of apical arms with crossed or split skeletal rods (arrows). (**g**) Deformed pluteus. (**h**) Percentages of anomalous plutei after the different experimental treatments. (**, *p* < 0.01; ***, *p* < 0.001; ****, *p* < 0.0001 with respect to control).

Observation under the microscope demonstrates that the morulae tend not to show significant morphological alterations. Only occasionally, at the highest DLZ concentration, blastomeres appeared dissociated, although surrounded by a vitelline membrane (Figure 4a) or, more rarely, morulae were completely altered (Figure 4b). For the plutei morphology, typical developmental alterations were observed: deformed (Figure 4c) or bent arms (Figure 4d) and abnormal positioning of skeletal rods (Figure 4e,f). Very rarely, completely deformed plutei were observed (Figure 4g). In controls, such alterations occurred in about 4.1 ± 0.9% of the plutei. In plutei obtained from pre-treated eggs, percentages were significantly reduced (maximum value 2.4 ± 0.7%); in contrast, in the other treatments, a significant increase in such percentages was observed at all DLZ concentrations. The maximum value (21.0 ± 2.3%) was registered in plutei obtained from sperm pre-treated with DLZ 5 µg/L, and the minimum value (7.7 ± 1.4%) in plutei treated at fertilization with DLZ 5 µg/L (Figure 4h).

**Figure 5:**
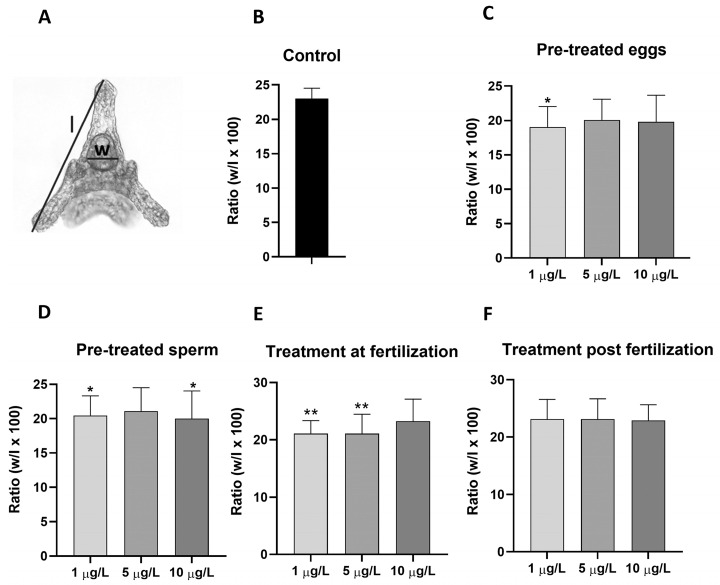
Effects of DLZ on gut development. (**A**) Pluteus with intact gut. Animal length (l) and gut width (w) were determined and used to identify possible anomalies in gut development. (**B**) Ratio (expressed as w/l × 100), in control samples. (**C**–**F**) Ratios after treatments. Notice moderate effects after gamete pre-treatments (**C**,**D**), more significant effects after exposure at fertilization (**E**), and the absence of effects after exposure during development (**F**). (*, *p* < 0.05; **, *p* < 0.01 with respect to control.)

In the different treatments, no significant morphological anomalies were detected in gut anatomy (Figure 5A). The relative size of the gut, in contrast, showed marked differences that depended on the moment exposure occurred (pre-treatment of eggs or sperm, exposure at fertilization or during development) but not on DLZ concentration. In control plutei, gut width was 23% of body length (Figure 5B). In plutei obtained from pre-treated eggs, the percentage dropped significantly (*p* < 0.05) to values around 19% without significant dose-dependent effects (Figure 5C). A similar situation was observed for plutei obtained from pre-treated sperm (Figure 5D). In this case, values reached a minimum percentage of 20% at DLZ 10 µg/L. In samples exposed at the time of fertilization, the effects seemed to add up (Figure 5E), while in the case of exposure after fertilization, no significant differences were observed in the ratio (Figure 5F).

### 2.4. Alterations in Carbohydrate Presence and Localization

(a) Morula stage (6 hpf)

**Figure 6:**
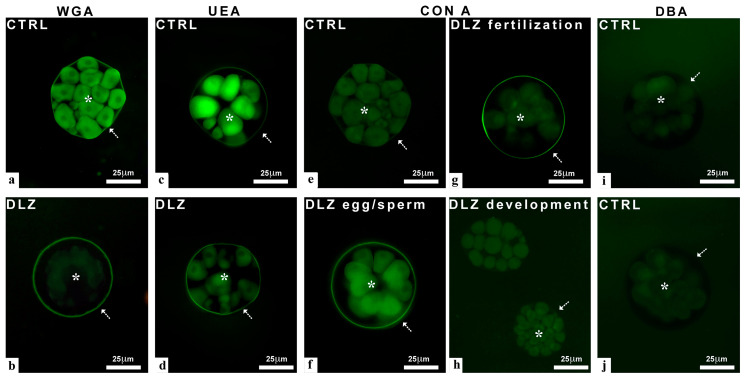
Effects of DLZ on carbohydrate presence and distribution in the morulae (6 hpf). Staining with FITC-lectins; blastomeres (*), fertilization membranes (arrows). (**a**) Morula with stained blastomeres and fertilization membrane. (**b**) Unstained blastomeres and stained fertilization membrane. (**c**) Stained blastomeres and fertilization membrane. (**d**) Moderately stained blastomeres and stained fertilization membrane. (**e**) Unstained morula. (**f**–**h**) Treatment-dependent staining of blastomeres and fertilization membranes. (**i**,**j**) Unstained morulae. WGA stains terminal N-acetyl glucosamine, UEA-I stains α-linked fucose, Con A stains α-linked mannose, DBA and SBA stain α- or β-linked N-Acetyl galactosamine. Bars: 25 µm.

In the control samples, the lectins WGA (Figure 6a) and the UEA-1 (Figure 6c) stained the cytoplasm of the blastomeres and the fertilization membrane. After exposure to DLZ, no matter the type of treatment or the concentration, the staining of the fertilization membrane was maintained (Figure 6b,d), while the staining of the blastomeres either disappeared completely (WGA, Figure 6b) or reduced significantly (UEA-1, Figure 6d). Control morulae (Figure 6e) and morulae exposed to DLZ during development (Figure 6h) were unstained by Con A. After treatment with DLZ, labeling appeared, differently distributed depending on the type of treatment but not on the concentration. In particular, in morulae obtained from pre-treated gametes, both blastomeres and fertilization membranes were labeled (Figure 6f), while in morulae exposed at fertilization, only the fertilization membrane was labeled (Figure 6g). Lectins DBA (Figure 6i) and SBA (Figure 6j) never stained the morulae, either controls or those treated with DLZ.

(b) Pluteus stage (48 hpf)

**Figure 7:**
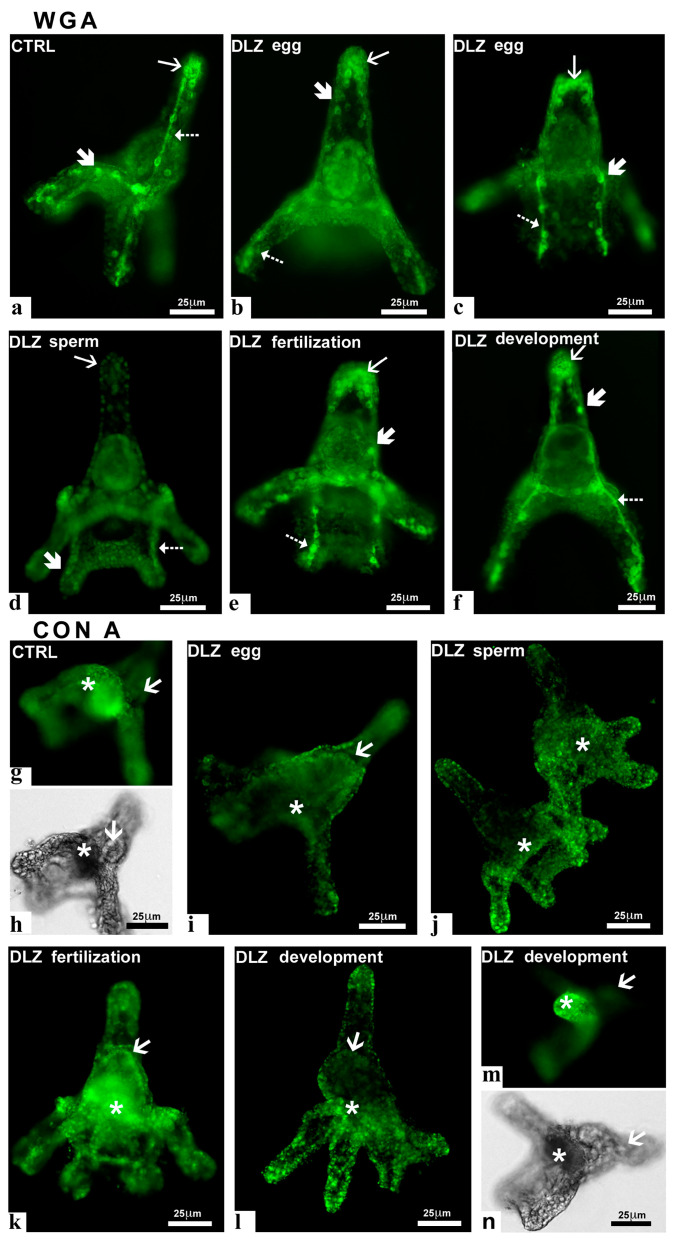
Effects of DLZ on carbohydrate presence and distribution in the plutei (48 hpf). Staining with FITC-WGA (**a**–**f**); skeletal rods (dotted arrows), mesenchyme cells (thick arrow), apical mesenchyme cells (thin arrow). (**a**) Pluteus with stained rods and mesenchyme cells. (**b**) Poorly stained rods and mesenchyme cells. (**c**) Intensely stained rods and mesenchyme cells in an abnormal 2-arm pluteus. (**d**) Unstained pluteus. (**e**,**f**) Stained skeletal rods and mesenchyme cells. Staining with FITC-Con A; stomodeum (*), gut (arrow). (**g**,**h**,**k**,**m**,**n**) Stained stomodeum and unstained gut. (**i**,**j,l**) Unstained stomodeum. WGA stains terminal N-Acetyl glucosamine and Con A stains α-linked mannose. Bars: 25 µm.

In control plutei, the WGA lectin intensely stained the skeletal rods and primary mesenchyme cells responsible for their production. These cells are located along the rods and at the apical end of the pluteus (Figure 7a). In plutei obtained from pre-treated eggs, labeling was either reduced (Figure 7b) or preserved (Figure 7c), without apparent correlation with the pluteus stage or condition. In contrast, in plutei obtained from pre-treated sperm (Figure 7d), no significant labeling was observed on rods or mesenchyme cells. In plutei treated at fertilization (Figure 7e) or during development (Figure 7f), no differences were observed with respect to the control. In all samples, no significant variations were observed based on DLZ concentration. In control (Figure 7g,h) plutei and in plutei exposed at fertilization (Figure 7k), the Con A lectin stained the stomodeum but not the gut. In plutei obtained from pre-treated eggs (Figure 7i) or pre-treated sperm (Figure 7j), no labeling was observed. In these cases, no significant differences were observed at the different DLZ concentrations. In contrast, in plutei exposed during development, no labeling was present after exposure to 1 or 5 µg/L (Figure 7l), while those exposed to 10 µg/L showed a labeled stomodeum (Figure 7m,n). Lectins DBA, SBA, and UEA-1 did not stain the plutei, indicating the absence of a significant amount of α- or β-linked N-Acetyl-galactosamine or α-linked fucose.

## 3. Discussion

The evidence collected demonstrates that DLZ impacts reproduction in the model organism *Paracentrotus lividus.* Concentrations close to those found in the environment reduce gamete quality and interfere with fertilization and development progression. The effects are fast, and the responses are not dose-dependent: apart from fertilization rate, all concentrations induced comparable effects on embryo development. Pre-treatment of gametes, like exposure at fertilization, drastically decreases the fertilization rate—an effect well in accord with observations in rats [39] but not in teleosts [17]. The effect is exerted only at the highest concentration, but the result is very significant: in waters, many different benzodiazepines are simultaneously present, and concentrations and effects of active principles and/or metabolites may be additive.

Regarding the site of action of DLZ, in the sperm, the tail axoneme and mitochondria are two main targets [40,41]. In the sea urchin, diazepam causes microtubule disassembly [40] and, by binding to the peripheral receptors TSPO (Translocator Protein, formerly Peripheral Benzodiazepine Receptor PBR), induces oxidative stress [41]. In addition, in mammals including humans, benzodiazepines can affect sperm capacitation [42] and the acrosome reaction, a process mediated by GABAergic receptors [43] and, by partitioning into the biological membranes [44], modify their fluidity [45]. In the sea urchin, the female gamete lacks centrosomes (carried along by the sperm [46]) and, therefore, organelles other than centrioles might be the target(s) of DLZ. Microtubules are improbable candidates, since after meiosis, their number and length gradually diminish [47]. Whatever the target site is, benzodiazepines alter folliculogenesis and affect meiosis progression, eventually reducing egg quality in mammals [48] or egg number, as reported in the gastropod *Radix balthica* [20]. In the sea urchin, at fertilization, DLZ can directly affect microtubule formation, causing atypical asters [40] or blocking the calcium-dependent exocytosis of cortical granules [49].

In the unfertilized sea urchin egg, it is difficult to propose a site of action for DLZ, since the gamete is dormant [50], the GABAergic system is lacking [51], and the mitochondrial activity is reduced to basal metabolism [52]. Mitochondrial permeability transition pores, however, are present and active [53] and an effect on the associated TSPO receptor on the mitochondrial membrane cannot be excluded. This point, unclear now, deserves further study, as many species lay eggs directly into water contaminated by high levels of benzodiazepines and the effects on fecundity may be relevant. As expected, the reduced fertility rate at 10 µg/L is followed by a decreased rate of morulae formation. These are normal from an anatomical point of view but show altered presence and/or distribution of sugar residues.

No matter if obtained from pre-treated gametes or a fertilization exposure, blastomeres lose N-Acetyl-glucosamine and α-linked fucose; meanwhile, only in the morulae derived from pre-treated gametes, there is an increase in α-linked mannose in both blastomeres and the fertilization membrane. In the sea urchin, carbohydrates are essential for cellular interactions and, among these, both fucose and mannose play an important role in sperm–egg interaction [54,55] and in early embryogenesis, as demonstrated by the disrupting effect of exposure to inhibitors of glycoprotein/proteoglycan synthesis [56] or binding to specific lectins [57]. Particularly significant is the fact that sugar variations are independent of DLZ concentration; in fact, they are manifest also in 1 and 5 µg/L samples, those in which fertilization percentage was as in controls.

The damages induced by early exposure to DLZ emerge clearly at the pluteus stage in the form of accelerations/delays in development. Interestingly, pre-treating eggs delays development (two-arm plutei are most abundant) while treatment at fertilization produces the opposite effect, anticipating development (more four-arm plutei). Interestingly, pre-treatment of sperm causes either a delay or an acceleration based on the DLZ concentration. The greater and smaller effects are exerted at the intermediate and higher concentrations, respectively, and as postulated in *Xenopus* [6], this response suggests the activation of detoxifying pumps when DLZ reaches a “critical” concentration. The ABC transporters in sea urchins are present in different forms, in male and female gametes and embryos [58], thus providing a basis to justify the different responses observed.

Putting together the morphological damage with the evidence obtained with lectin staining is not easy. N-acetyl glucosamine plays a role in the positioning of primary mesenchyme cells [59]; therefore, the reduced labeling for WGA in the blastomeres suggested that a high percentage of plutei would show damaged skeletal rods. This however occurs only in plutei obtained from pre-treated sperm. In contrast, the increase in α-linked mannose in morulae obtained from pre-treated gametes perfectly fits with the observed damage in the stomodeum, indicated by the lack of Con A positivity, and with the reduced gut width. Together with the interferences with developmental times, the absence of Con A staining suggests that DLZ exerts teratogenic effects, in particular on the gut, confirming recent evidence on *Xenopus laevis* embryos, in which the same concentrations of DLZ used in this study caused gut immaturity [6] and perturbation in DNA methylation [60]. 

The results obtained by treatment during development are quite different from those recorded after pre-treating gametes or treatment at fertilization. The effects appear early but are registered at all concentrations: 6 hpf, only 50% of the embryos had reached the morula stage. GABA and TSPO receptors are present in the embryos, as neurotransmitters regulate morphogenetic activities such as proliferation, differentiation, and cell motility [61]. Delay, therefore, can be attributed to interference of DLZ with mitosis, to anomalies in spindle formation [62], or alteration of centrosomal material and/or in microtubules [63]. Similar effects have been demonstrated in tumor cells—systems that, like embryos, show intense and rapid mitotic activity [64].

In this case, too, the morulae do not show significant macroscopic alterations but significant variations in carbohydrate residue’s presence and distribution occur in both blastomeres and fertilization membranes. In echinoderm embryos, from the 16-cell stage, glycoproteins are exposed on the surface of the plasma membranes to modulate cell adhesion and recognition [65]. These are both derived from the maternal pool [66] and synthesized from blastomeres by glycosyltransferases [65]. The observed reduced affinity for lectins suggests a potential interference of DLZ with the activity of these enzymes but at the moment, the only support for this hypothesis comes from a report demonstrating that diazepam alters glycosyltransferase activity in a plant [67]. Proteoglycans are necessary also for post-gastrula development [68] and the relatively modest percentage of altered plutei seems to confirm that the embryos have indeed recovered from the initial damage observed at the morula stage. The percentage of two- and four-arm plutei is as in controls, and the gut appears normally developed. 

Put together, the data collected suggests that DLZ can impact several processes and mechanisms in *Paracentrotus* gametes and early embryos. This would depend on the different presence and distribution of the two primary targets of benzodiazepines: GABA and TSPO receptors. The effects of these drugs on male and female gametogenesis have already been reported [15,16] but the molecular mechanisms at the base remain elusive.

## 4. Materials and Methods

### 4.1. Animal Handling

*Paracentrotus lividus* (*Strongylocentrotus lividus*, Lamarck, 1816), a Mediterranean species common along the Italian coasts, was provided by local suppliers. Once acclimated for 48 h, it was maintained in 25 L aquaria filled with seawater (Instant Ocean Sea Salt, Blacksburg, VA, USA), salinity 36 ± 1‰ g/L, with constant aeration, according to Giudice [69]. All the experiments were conducted at 18 °C under a 12 h light (12 h dark photoperiod).

### 4.2. DLZ Solutions Preparation

For the treatment, a largely consumed benzodiazepine preparation in the form of oral drops was used. It contains the active principle DLZ at 1 mg/mL concentration and excipients in unspecified quantities [6]. The operating solution was prepared by diluting the preparation in artificial seawater to a final concentration of 1, 5, or 10 µg/L of DLZ [6,7]. The first concentration was chosen based on the environmental concentrations reported in the literature for the aquatic environments, calculated considering the average concentration of different benzodiazepines in European waste and coastal waters [6,7,70,71,72]. The two latter concentrations were chosen considering the growing trend of benzodiazepine (ab)use and the fact that more than one benzodiazepine is present simultaneously in the same environment, thus suggesting the occurrence of additive effects.

### 4.3. Fertilization and Larval Growth

The method refers to what is described in Giudice et al. [69]. For each experiment, gametes were obtained through intracoelomic stimulation using 0.5 M KCl from four distinct animals and then pooled. The eggs were released into seawater, while the sperm was preserved dry in a Petri dish placed on ice. Before utilization, the quality of the gametes was assessed using a microscope: aliquots of eggs were examined for their shape and appearance, and 5 μL of sperm was activated in seawater and checked for number, morphology, and motility. This preliminary assessment facilitated the creation of culture samples with the appropriate number of eggs and sperm. In each experimental trial, around 7200 eggs were subjected to a thorough wash in clean water to eliminate debris, and then randomly divided to establish triplicate cultures for control and DLZ concentrations. Fertilization was induced by introducing a suitable volume of activated sperm (maintaining an approximate sperm–egg ratio of 1500:1). The growth phase occurred in sterile glass Petri dishes (25 cm in diameter) containing approximately 600 embryos in 150 mL of seawater. Cultures were consistently agitated on a tilting laboratory shaker and discarded if the fertilization percentage in the control group fell below 95%.

### 4.4. Treatments

**Figure 8:**
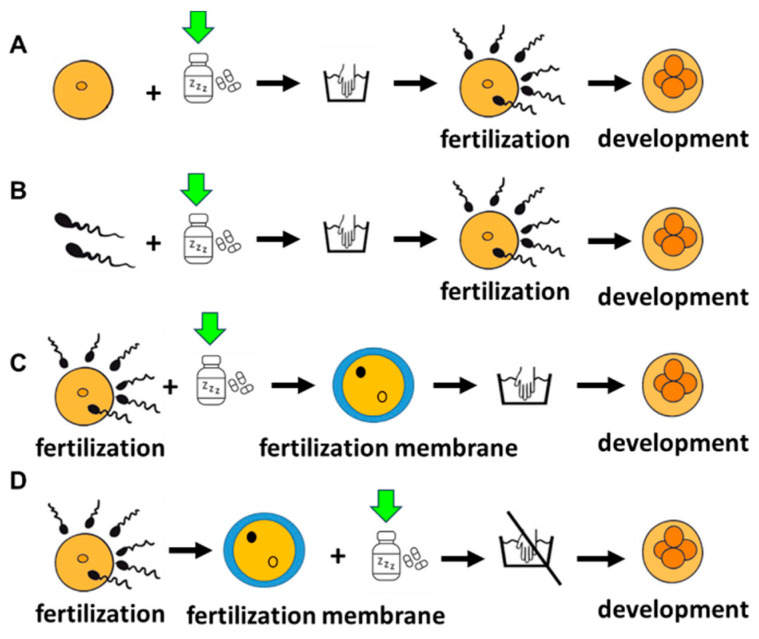
Schematic representation of the four treatments carried out. (**A**,**B**) Eggs and sperm pre-treatment with DLZ; washing and use in fertilization with untreated sperm or eggs. Development in pure seawater. (**C**) Fertilization of untreated gametes in the presence of DLZ; washing after the elevation of the fertilization membrane and development in pure seawater. (**D**) Fertilization of untreated gametes in pure seawater; addition of DLZ after the elevation of the fertilization membrane and development in the presence of DLZ.

To verify the interference of DLZ with fertilization, eggs or sperm were either pre-treated and then used for fertilization with untreated sperm or eggs, respectively, or treated directly at the time of fertilization. In brief, the eggs were exposed for 20 min to the DLZ solutions, gently washed in clean seawater, inseminated with untreated sperm, and allowed to complete development in clean seawater (Figure 8A). Sperm were activated in seawater containing DLZ and used to fertilize untreated eggs (Figure 8B). For the exposure at the fertilization time, sperm and DLZ were simultaneously added to the egg culture; twenty minutes after the first fertilization membrane appeared, all the eggs were collected using a nytex membrane (100 µm pores), gently washed in clean seawater to eliminate debris and sperms, and transferred into the Petri dish containing pure seawater where they were allowed to complete development (Figure 8C).

To verify the effects of exposure on development, the eggs were fertilized in uncontaminated seawater using untreated sperm. Twenty minutes after the first fertilization membrane appeared, the fertilized eggs were collected, resuspended in seawater containing DLZ, and allowed to complete development (Figure 8D).

### 4.5. Sampling and Data Analyses

Development was followed at 6 hours (morula stage) and 48 hours (pluteus stage) post fertilization (hpf [73]) by collecting aliquots of the embryo culture. Morulae and plutei were fixed in 4% formaldehyde [74] and thoroughly washed in 75% ethanol at the time of observation under the microscope. At least 600 larvae were observed at each time point for each treatment; the experiments were carried out in triplicates. Percentages of fertilization were determined under a Leica MZ16 A stereomicroscope (Leica Microsystems s.r.l., Milan, Italy) by counting the number of eggs that showed a fertilization membrane out of the total number of eggs treated × 100. Larval anomalies (delays in the stage of development and/or alteration in shape) were also recorded. For plutei, gut anatomy was assessed by determining the relative size of the stomach. Images were acquired using a Zeiss Axiocam camera applied to a Zeiss Axioskop microscope (Zeiss, Oberkochen, Germany) and then analyzed using ImageJ (free version 1.8.0; last update 22 May 2023; https://imagej.net/ij/download.html) to determine maximum arm length (AL) and stomach width (SW) [75].

### 4.6. Identification of Carbohydrate Residues by Staining with Fluorescent Lectins

Morulae and plutei fixed in 4% formaldehyde were washed in seawater and 50% ethanol. Small aliquots of the suspension (150 µL, containing approximately 100 morulae or plutei) were transferred into a vial on ice, in the dark, and stained by adding 3 μL of one of the FITC-lectins (Vector Laboratories Inc, Newark, CA; 2 mg/mL). In particular, WGA was used to stain terminal N-Acetyl glucosamine (glcNAc), Con A to stain α-linked mannose, UEA-I to stain α-linked fucose, and DBA or SBA to stain α- or β-linked N-Acetyl galactosamine, respectively. Negative controls were prepared by incubating with the lectin and the specific competing sugar or by omitting the lectin to test potential autofluorescence [75]. After 2 h, aliquots were pipetted onto a slide, covered with a coverslip, and morulae or plutei were immediately observed under a UV microscope (excitation maximum at 495 nm and emission maximum at 515 nm) at constant exposure times. Labeling was defined as positive or negative by two independent observers. Negative controls were always completely unstained.

### 4.7. Statistical Analysis

Numerical data obtained for fertilization rate and growth progression at 6 and 48 h were normalized by applying the following formula: mean value of treatment sample/mean value of control sample × 100. To evaluate proper stomach development, the ratio of stomach width/animal length × 100 [76,77] was determined. All values obtained were analyzed for significance by One-Way ANOVA or the Student’s t-test using GraphPad-Prism 8 software (GraphPad Software, Inc., San Diego, CA, USA) [6]. For all tests, the minimum significance level accepted was *p* < 0.05. Cohen’s d was used to determine the size effects of treatments, in particular, d = (x1 − x2)/s, where x1 and x2 are the sample means of group one and group two, and “s” is the standard deviation of the population from which the two groups were taken [78].

## 5. Conclusions

In conclusion, this preliminary work demonstrates that environmental concentrations of a largely abused benzodiazepine, DLZ, significantly interfere with fertilization and larval development. The most evident effects are exerted on sperm (reduced fertilization rate and high percentage of altered plutei) and early cleavage (poor morulae development). Lectin staining highlights that the damage is far more extensive, affecting both morulae and plutei, no matter the drug concentration or the time of exposure. The sea urchin *Paracentrotus lividus* confirms an excellent model for reproductive studies and toxicity testing, and the results so far obtained will pave the way for more specific and applicative research. In particular, it will be essential to investigate whether DLZ interferes with gene expression in this species as well as in other non-target species. Site and mechanisms of action should be clarified, considering that interference is also potentially exerted in species with internal fertilization, humans included.

## Data Availability

Data are contained within the article.
